# Nonalcoholic Fatty Liver Disease-Associated Liver Fibrosis Is Linked with the Severity of Coronary Artery Disease Mediated by Systemic Inflammation

**DOI:** 10.1155/2021/6591784

**Published:** 2021-12-28

**Authors:** Ling-zi Chen, Xu-bin Jing, Chao-fen Wu, Yi-cheng Zeng, Yan-chun Xie, Mu-qing Wang, Wen-xia Chen, Xi Hu, Yan-na Zhou, Xian-bin Cai

**Affiliations:** ^1^Department of Gastroenterology, The First Affiliated Hospital of Shantou University Medical College, Shantou, Guangdong 515041, China; ^2^Department of Endoscopy Center, Shantou Central Hospital, Shantou, Guangdong 515041, China; ^3^Department of Endoscopy Center, Puning People's Hospital, Jieyang, Guangdong 515200, China

## Abstract

**Methods and Results:**

We conducted a retrospective study of 531 patients with ultrasonogram-confirmed NAFLD who underwent percutaneous coronary intervention (PCI). Then, all patients were separated into four categories by Gensini score (0, 0-9, 9-48, and ≥48) for use in ordinal logistic regression analysis to determine whether NAFLD fibrosis was associated with increased Gensini scores. Mediation analysis was used to investigate whether systemic inflammation is a mediating factor in the association between NAFLD fibrosis and CAD severity. FIB − 4 > 2.67 (OR = 5.67, 95% CI 2.59-12.38) and APRI > 1.5 (OR = 14.8, 95% CI 3.24-67.60) remained to be independent risk factors for the severity of CAD after adjusting for conventional risk factors, whereas among the inflammation markers, only neutrophils and neutrophil-to-lymphocyte ratio (NLR) were independently associated with CAD. Multivariable ordinal regression analysis suggested that increasing Gensini score (0, 0-9, 9-48, and ≥48) was associated with advanced NAFLD fibrosis. ROC curve showed that either fibrosis markers or inflammation markers, integrating with traditional risk factors, could increase the predictive capacity for determining CAD. Inflammation markers, especially neutrophils and NLR, were mediators of the relationship between NAFLD fibrosis and CAD severity.

**Conclusions:**

NAFLD patients with advanced fibrosis are at a high risk of severe coronary artery stenosis, and inflammation might mediate the association between NAFLD fibrosis and CAD severity.

## 1. Introduction

Nonalcoholic fatty liver disease (NAFLD) is becoming one of the most common chronic liver diseases, with prevalence up to 20%-30% around the world [1], and it is demonstrated to be a systemic disease rather than a simple hepatic disease. NAFLD encompasses a spectrum of liver diseases ranging from liver steatosis, nonalcoholic steatohepatitis (NASH) to liver fibrosis that may lead to cirrhosis and hepatocellular carcinoma [2]. The presence of liver fibrosis, which can be evaluated by liver biopsy, imaging technique, or noninvasive biomarkers, is the most important factor worsening the prognosis of NAFLD patients. Although liver biopsy is still the gold standard, fibrosis biomarkers which have been advocated in current guidelines to detect severe fibrosis in clinical practice due to its advantages of simplicity and noninvasion may reduce the need for liver biopsy by identifying NAFLD patients at high risk of advanced liver fibrosis [3].

Cardiovascular disease (CVD) is now considered as the primary cause of death in NAFLD patients, rather than liver-related complications. The NAFLD is not only a marker of coronary heart disease (CHD) but may also be involved in the pathogenesis of CHD which has been discussed [4]. Several prospective studies have detected a robust association between NAFLD and the presence and outcomes of CVD, independent of metabolism syndrome [5, 6]. Furthermore, NAFLD fibrosis detected by noninvasive biomarkers, such as NAFLD fibrosis score (NFS) and fibrosis-4 score (FIB-4), has been indicated to be associated with increased risk of cardiovascular events [7]. However, there is a lack of sufficient evidence on the association of NAFLD fibrosis with CAD severity. It is important to elucidate whether advanced liver fibrosis in NAFLD contributes to the progression of CVD, which could provide evidence that early intervention towards NAFLD may improve CVD prognosis. Whether NAFLD directly contributes to pathogenesis of CVD or increases CVD risk due to shared risk factors is unclear. Several potential pathophysiologic mechanisms are involved, such as dyslipidemia, insulin resistance, oxidative stress, low-grade systemic inflammation, and endothelial dysfunction [8]. Systemic inflammation has been indicated to be correlated with both NAFLD and CAD; however, whether inflammation acts as a shared risk factor or plays a bridge role in the relationship between NAFLD and CAD remains undefined.

In this study, we investigate the relationship between NAFLD fibrosis defined by noninvasive assessment and CAD severity detected by Gensini score and confirm the underlying mechanism involved in inflammation.

## 2. Methods

### 2.1. Study Population

**Figure 1:**
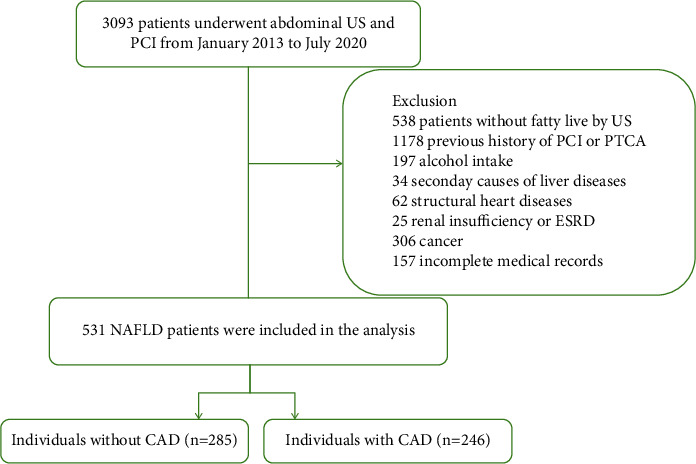
Flowchart of the population included in the study. US: ultrasonogram; PCI: percutaneous coronary intervention; PTCA: percutaneous transluminal coronary angioplasty; ESRD: end-stage renal disease; NAFLD: nonalcoholic fatty liver disease; CAD: coronary artery disease.

We conducted a retrospective, cross-sectional study on 3,093 patients who underwent abdominal ultrasonogram (US) and percutaneous coronary intervention (PCI) at the Department of Cardiology in the First Affiliated Hospital of Shantou University Medical College from January 2013 to July 2020 (Figure 1). Patients without evidence of fatty liver on US (*n* = 538) were excluded. We also excluded patients who met the following criteria: previous history of PCI or percutaneous transluminal coronary angioplasty (PTCA) (*n* = 1178), history of alcohol intake (*n* = 197), secondary causes of liver diseases (e.g., viral hepatitis or autoimmune liver disease) (*n* = 34), history of structural heart diseases (e.g., rheumatic heart disease, cardiomyopathy) (*n* = 62), renal insufficiency or end-stage renal disease (ESRD) (*n* = 25), infectious diseases (*n* = 65), previous history of cancer (*n* = 306), and incomplete medical records (*n* = 157). Finally, 531 patients were included in the study.

### 2.2. Clinical and Laboratory Data

Patients' demographic data, such as age and gender; smoking habits; and history of diabetes mellitus and hypertension were derived from electronic medical records. Laboratory data were also acquired when patients were admitted, including levels of blood cell count (neutrophils, lymphocytes, monocytes, and platelets); liver biochemistry (alanine aminotransferase (ALT) and aspartate aminotransferase (AST) levels, *γ*-glutamyl transpeptidase (GGT), bilirubin, and albumin); fasting glucose and glycated hemoglobin (HbA1c); parameters of renal function (serum creatinine, uric acid, and Cystatin C); and total cholesterol, triglycerides, low-density lipoprotein cholesterol (LDL), and high-density lipoprotein cholesterol (HDL). NLR, one of the inflammation markers, was calculated as the ratio of neutrophil to lymphocyte, the level of which was obtained from the same blood sample.

### 2.3. Assessment of the CAD Severity

CAD was defined as the presence of at least one of the main coronary arteries with ≥50% obstruction, including the left main coronary artery (LM), left anterior descending artery (LAD), left circumflex coronary artery (LCX), and right coronary artery (RCA), or main branches of the vascular system. The severity of coronary atherosclerosis is assessed based on the Gensini score, which is determined according to the severity of stenosis as follows: 1 point for less than 25% obstruction, 2 points for 26% to 50% obstruction, 4 points for 51% to 75% narrowing, 8 points for 76% to 90% narrowing, and 32 points for complete occlusion. Each lesion score is multiplied by a factor that considers the importance of the lesion's position in the coronary circulation (5 for LM; 2.5 for the proximal segment of LAD and LCX; 1.5 for the midsegment of LAD; 1.0 for any segment of RCA, the distal segment of LAD and LCX, the first diagonal artery, the posterolateral artery, and the obtuse marginal artery (OM); and 0.5 for other segments) [9]. We defined non-CAD patients as having Gensini score = 0, and all CAD patients were separated into 3 groups in accordance with the tertiles of Gensini score, including patients with mild stenosis (0 < Gensini score ≤ 9), moderate stenosis (9 < Gensini score < 48), and severe stenosis (Gensini score ≥ 48).

### 2.4. Noninvasive Fibrosis Marker

Noninvasive fibrosis markers, FIB-4 index and AST-to-platelet ratio index (APRI), were assessed in each NAFLD patient. These fibrosis markers were calculated using the following formula, respectively:

(1) $$\begin{array}{c} \text{FIB}‐4\text{ index}=\frac{\left[\text{age}\left(\text{years}\right)\times \text{AST}\left(\text{IU}/\text{L}\right)\right]}{\left[\text{platelet counts }\left(\times 10^{9}/\text{L}\right)\times {\left(\text{ALT }\left(\text{IU}/\text{L}\right)\right)}^{1/2}\right]}, \end{array}$$

(2) $$\begin{array}{c} \text{APRI}=\left[\frac{\left(\text{AST}/\text{ULN}\right)}{\text{platelet counts }\left(\times 10^{9}/\text{L}\right)}\right]\times 100*\text{ULN }\left(\text{ULN means the upper level of normal}\right). \end{array}$$

The cut-off value of 2.67 and 1.5 (for FIB-4 and APRI, respectively) was applied to determine high probability of advanced fibrosis of NAFLD [10, 11].

### 2.5. Statistical Analysis

Statistical analyses were performed by using the SPSS version 25.0 software. Continuous variables are presented as mean ± standard deviation (SD) or median (IQR) and compared between patients with and without CAD. Categorical variables are presented as percentages. For the univariate analysis between CAD and non-CAD, continuous variables were compared using unpaired *t* test or Mann-Whitney test as appropriate, and categorical variables were compared using the chi-square test or Fisher's exact test. Binary logistic regression analysis was performed to identify independent factors associated with CAD, and ordinal regression analysis was used to examine the association between fibrosis markers and severity of CAD. Variables with a *p* value less than 0.05 in the univariate logistic regression analyses were included in the multivariate logistic regression analysis. Receiver operating characteristic (ROC) curves were constructed to assess the predictive value of fibrosis markers and inflammation markers for CAD. Mediation analysis was accomplished by SPSS version 25.0 software, using the PROCESS macro (Process V3.2) developed by Hayes and Preacher [12]. Figure S1 is a mediation analysis path diagram. A *p* value less than 0.05 was considered statistically significant.

## 3. Results

### 3.1. Baseline Characteristics

The baseline clinical characteristics, laboratory data, and fibrosis markers of patients with versus without CAD are presented in Table 1. As presented in Table 1, the mean age of CAD patients was 63 ± 10 years, and that of the non-CAD patients was 60 ± 9 years (*p* < 0.001). CAD patients were more likely to be male, with smoking habits, hypertension, and DM. Inflammation marker levels, such as neutrophil, monocyte, and NLR, were notably higher in the CAD group than those in the non-CAD group, while HDL was lower in the CAD group. Similarly, other serum markers, e.g., AST, total protein, albumin, creatine, GLU, HbA1c, and Cys C, were higher in CAD. On the other hand, FIB-4 and APRI were both higher in CAD patients than in non-CAD patients. There were no significant differences in lymphocyte, platelet, ALT, GGT, TBIL, DBIL, cholesterol, triglyceride, and LDL between the two groups (*p* > 0.05).

### 3.2. Factors Associated with Coronary Artery Disease

The results of the multivariate logistic regression analysis (Table S1) suggested that only older age, male, history of hypertension and DM, higher AST, and lower level of HDL remained as independent factors associated with coronary artery disease. To assess the association between CAD and noninvasive fibrosis markers and inflammation markers, respectively (Table 2), we performed multivariate analysis adjusted for well-established risk factors, such as gender, age, smoking history, hypertension, DM, triglyceride, HDL, and LDL. FIB-4 and APRI both remained to be independent risk factors for CAD (OR = 5.67, 95% CI: 2.59-12.38, *p* < 0.001, and OR = 14.81, 95% CI: 3.24-67.6, *p* = 0.001, respectively). Similarly, among inflammation markers, only neutrophil and NLR were independently associated with coronary artery disease after adjustment (OR = 1.35, 95% CI: 1.21-1.50, *p* < 0.001, and OR = 1.18, 95% CI: 1.08-1.30, *p* < 0.001, respectively).

### 3.3. Noninvasive Fibrosis Markers, Inflammation Markers, and Gensini Score

Table 3 illustrates the relationship between fibrosis markers and Gensini score. The odds ratio (OR) for different fibrosis markers associated with one step increase was statistically significant between severity categories, including that from the ordinal regression analysis (OR = 3.33, 95% CI: 2.42-4.60, *p* < 0.001, and OR = 4.65, 95% CI: 2.92-7.43, *p* < 0.001, respectively). The relationship between invasive fibrosis markers and Gensini score was still significant but attenuated when other classical risk factors were considered.

### 3.4. Association between Inflammation Markers and Gensini Score

The distribution of inflammation markers was examined according to Gensini score (Figure S2). Apparently, neutrophil counts were significantly higher in patients with severe coronary artery stenosis than in those with lower Gensini score. Similarly, monocyte counts and NLR were both greater in patients with higher Gensini score. There was not statistically significant between lymphocyte counts and Gensini score.

### 3.5. ROC Curve Analysis of Fibrosis Markers and Inflammation Markers

**Figure 2:**
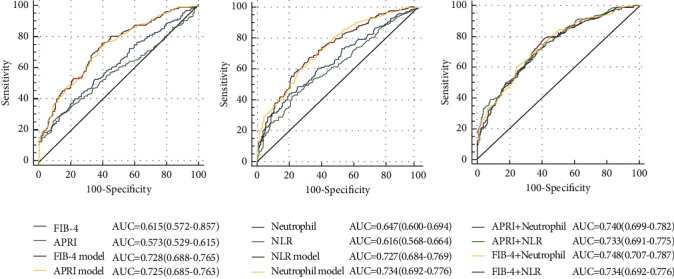
Receiver operating characteristic (ROC) curve analysis of the predictive power of fibrosis markers, NLR, and neutrophil for CAD. APRI model, FIB-4 model, NLR model, neutrophil model, APRI+neutrophil, and FIB-4+neutrophil: new models integrating noninvasive markers and recognized risk factors for CAD (gender, age, smoking, hypertension, DM, triglyceride, HDL, LDL).

The performance of noninvasive markers for predicting CAD was evaluated by ROC curves (Figure 2). The AUROCs (95% CI) for the FIB-4, APRI, neutrophil, and NLR were 0.615 (0.572-0.657), 0.573 (0.529-0.615), 0.647 (0.600-0.694), and 0.616 (0.568-0.664), respectively. After integrating well-established risk factors, the AUROCs for these models increased to 0.728, 0.725, 0.734, and 0.727, respectively, which were greater than those of original models. Further, when integrating fibrosis markers and neutrophil and NLR with classical risk factors, the AUROCs were slightly increased.

### 3.6. Mediating Effect Analysis on the Association between Fibrosis Markers and Gensini Score

**Figure 3:**
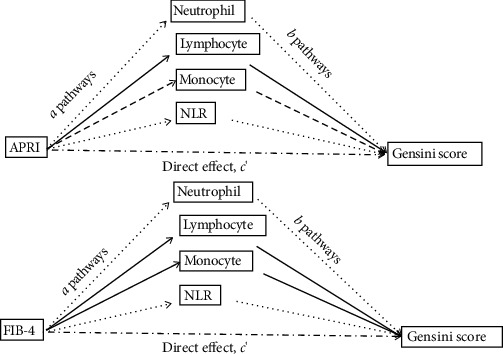
Mediated model of fibrosis markers with Gensini score. [1] The coefficient *a* is the effect of fibrosis markers on the inflammation markers; [2] the coefficient *b* is the effect of inflammation markers on Gensini score after controlling the influence of fibrosis markers; [3] the coefficient *c*′ is the direct effect of fibrosis markers on Gensini score after controlling the influence of inflammation markers. Dash line means the pathway is statistically significant.

The mediating effects of inflammation markers on the association between fibrosis markers and Gensini score are presented in Table 4. The first column, which showed the relative effects of FIB-4 and APRI on inflammation markers, suggested that FIB-4 were significantly associated with neutrophil, lymphocyte, and NLR, while APRI were significantly associated with all mediators. The relative direct effects on Gensini score were demonstrated in the second column. In the mediated model of FIB-4, only neutrophil and NLR significantly correlated with Gensini score. It showed that the 95% bootstrapped CI for the indirect effects of neutrophil (95% CI, 0.696-2.080) and NLR (95% CI, 0.112-1.542) on Gensini score were significantly different from zero. Similarly, in the APRI model, the 95% bootstrapped CI for the indirect effects of inflammation markers (except for lymphocyte) on Gensini score were significantly different from zero. In conclusion, to some extent, inflammation markers, especially neutrophil and NLR, can explain the association between NAFLD fibrosis and CAD severity. The mediated models depicted in Figure 3 indicate that the association between fibrosis markers and Gensini score is both directly via the *c*′ pathway (relative direct effects on Gensini score) and indirectly through the mediated *a*∗*b* pathway (relative indirect effects on Gensini score).

## 4. Discussion

In this study, the main findings are as follows: [1] FIB-4 and APRI were both independently correlated with the presence and severity of CAD in NAFLD and [2] chronic inflammation may mediate the association between NAFLD fibrosis and CAD severity.

A growing body of evidence shows that the presence of NAFLD is associated with an increasing prevalence and incidence of cardiovascular disease, independent of established cardiovascular risk factors, such as obesity, hypertension, diabetes, smoking history, age, and hyperlipidemia [13]. Furthermore, some studies have further investigated the relationship between progression of NAFLD and CAD outcomes, and inconsistent results were obtained. A recent meta-analysis of 16 observational studies by Targher et al. reported that patients with severe NAFLD, characterized by presence of hepatic steatosis on imaging plus either elevated GGT levels, high NFS, high hepatic FDG uptake on PET-CT, or advanced fibrosis stage on liver biopsy, were at a higher risk for cardiovascular events [13]. In a prospective cohort study of 11,154 subjects, Kim et al. showed that NAFLD detected by USG did not increase the risk of mortality, whereas NAFLD with evidence of advanced fibrosis, as defined by noninvasive fibrosis markers, was associated with mortality mainly from cardiovascular causes [14]. However, studies discussing about the relationship between CAD severity and progression of NAFLD are limited. Only a few studies indicated that severe NAFLD determined by ultrasonography independently increased the risk for severe CAD assessed by coronary artery obstruction, Gensini score, and multivessel disease on angiography [ 15–17]. In our analysis, we determined the degree of liver fibrosis by noninvasive score system rather than ultrasonography which is a highly operator-dependent procedure with inter- and intraobserver variability. It is well-known that liver biopsy is the gold standard for the diagnosis and grading of liver fibrosis; however, it is difficult to apply in clinical practice due to its limitation including sample error, serious complication, and low patient acceptance. Serum biomarkers have the advantages of low cost, repeatability, and high applicability (>95%). Diagnostic accuracy of FIB-4 is superior to other simple noninvasive tests such as NFS, APRI, and BARD score, and the summary specificities of APRI and FIB-4 were both greater than 85% for predicting severe fibrosis [18]. Furthermore, noninvasive detection of liver fibrosis allows the dynamic monitoring of liver function, which has great value in prognosis assessment [19].

In the present study, we used coronary angiography to depict the presence of coronary stenosis and assess the CAD severity by Gensini score. We found that FIB-4 and APRI were both independently associated with the presence and severity of CAD after correcting for traditional risk factors; that is, NAFLD patients with advanced fibrosis were more likely to suffer from severe coronary artery stenosis. Moreover, ROC analysis suggested that fibrosis markers could obviously improve the predictive power in diagnosing CAD when integrating traditional risk factors. Hence, fibrosis markers may become useful tools for predicting the presence and severity of CVD in clinical practice.

Hepatic steatosis may progress to hepatocyte injury and initiate the inflammation, which may aggravate insulin resistance, glucose intolerance, and excessive accumulation of lipids in the liver [20]. Accumulation of inflammation mediators to some extent affects the onset, progression, and outcomes of cardiovascular disease. Emerging evidence has demonstrated that the imbalance of immunologic homeostasis in the liver during the progression of NAFLD may lead to systemic inflammation, which directly contributes to cardiovascular diseases [21]. Some evidence showed that parameters of white blood cells (WBC) and their subtypes such as neutrophils and lymphocytes were associated with adverse outcomes or mortality in patients with cardiovascular disease [22, 23]. Emerging knowledge suggests that neutrophils not only initiate atherosclerosis but also promote atherosclerotic plaque instability, leading to the various stages of atherosclerosis and clinical manifestations of CAD, including myocardial infarction, heart failure, and stroke [24]. In fact, NLR is becoming a widely available marker for predicting the severity and major clinical adverse outcomes of CAD [25, 26]. The association between NLR and CVD can be explained by various possible mechanisms. Neutrophils secrete inflammatory mediators that can facilitate plaque disruption [27], whereas lymphocytes regulate the inflammatory response and play an antiatherosclerotic role [28]. In our study, neutrophils and NLR were both associated with the presence and severity of CAD evaluated by Gensini score, which was consistent with previous studies [26, 29, 30].

In our study, inflammation markers were associated with CAD severity. It should be noted that chronic inflammation may not only be shared risk factor but also act as mediators of the relationship between NAFLD and CVD. NASH accompanied with a systemic, low-grade inflammation promotes the progression of CAD [31]. To further explore the mediation effect of inflammation markers on the pathway between NAFLD fibrosis and Gensini score, we applied a mediation analysis—a more sophisticated analytical method. It is indicated that neutrophils played a partial mediating role in the relationship between fibrosis and CAD severity, which had the strongest mediating effect compared to other inflammation markers. In fact, mediation analysis is commonly used in psychology and sociology to investigate the mechanism of interventions, which is rarely used in cardiovascular research. To the best of our knowledge, this is the first study discussing the mediating effect of chronic inflammation in the relationship between NAFLD fibrosis and CAD severity. Our results suggested that NAFLD fibrosis is associated with the progression of CAD via the mediation of systemic inflammation.

There are several limitations in this study. First, this is a single-center retrospective research that includes a relatively small number of patients and lacks follow-up data, which could not provide a causal relationship or prognostic significance of NAFLD fibrosis in the progression of CAD. Therefore, our findings need to be confirmed in other larger-sample prospective studies with long-term clinical follow-up. Second, NAFLD was diagnosed by ultrasonogram which is not high-sensitive tool detecting fatty liver with more than 30% steatosis. However, ultrasonography has the advantages of safety, low cost, and repeatability, which could be a convenient imaging technique for diagnosing NAFLD in clinical practice. Third, since an alcohol consumption history could not be obtained quantitatively, it was difficult to fully discriminate between alcoholic fatty liver disease (AFLD) and NAFLD; thus, we excluded patients with alcohol intake history, which may lead to the decrease in sample size. Finally, we could not explore the underlying mechanism in the relationship between NAFLD fibrosis and CAD severity, because mediation analysis can only verify the possible causal hypothesis. In conclusion, further studies are needed to investigate and validate the underlying mechanism of the association between NAFLD fibrosis and cardiovascular disease.

## 5. Conclusion

In conclusion, the present study suggested that NAFLD with advanced fibrosis is associated with severity of CAD. Moreover, systemic inflammation might play a mediating role in the association between NAFLD fibrosis and CAD severity. FIB-4 and APRI, as simple, available, and inexpensive biomarkers, can help identify NAFLD patients at high risk for severe CAD, which can be used as convenient tools in clinical practice. Therefore, it is warranted that the early intervention towards fatty liver to prevent the progression of cardiovascular disease.

## Figures and Tables

**Table 1:** Baseline characteristics of NAFLD patients with and without CAD.

Variables	Non-CAD (*n* = 285)	CAD (*n* = 246)	*p* value
Clinical characteristics			
Age (year)	60 ± 9	63 ± 10	<0.001
Male, *n*(%)	96 (33.7)	141 (57.3)	<0.001
Smoking, *n*(%)	50 (17.5)	91 (37.0)	<0.001
Hypertension, *n*(%)	147 (51.6)	163 (66.3)	0.001
DM, *n*(%)	70 (24.6)	90 (36.6)	0.003
Laboratory data			
Neutrophil (10^9^/L)	4.36 ± 1.56	5.57 ± 2.54	<0.001
Lymphocyte (10^9^/L)	2.13 ± 0.69	2.07 ± 0.76	0.37
Monocyte (10^9^/L)	0.49 ± 0.17	0.55 ± 0.21	<0.001
NLR	2.40 ± 1.99	3.28 ± 2.75	<0.001
Platelet (10^9^/L)	230 ± 54	231 ± 57	0.88
AST (U/L)	23 (18, 29)	24 (19.0, 40.1)	0.006
ALT (U/L)	24 (17.0, 37.5)	26 (18.0, 39.2)	0.201
GGT (U/L)	32 (22.4, 50.0)	33 (25.4, 51.2)	0.225
Total protein (g/L)	71.33 ± 7.69	69.86 ± 6.29	0.02
Albumin (g/L)	40.41 ± 3.63	39.32 ± 3.75	<0.001
TBIL (*μ*mol/L)	13.71 ± 5.86	13.72 ± 6.56	0.98
DBIL (*μ*mol/L)	2.45 ± 1.98	2.49 ± 1.32	0.75
GLU	6.06 ± 1.57	6.99 ± 2.66	<0.001
HbA1c (%)	6.28 ± 1.01	6.82 ± 1.39	<0.001
Creatinine (*μ*mol/L)	86.81 ± 18.77	97.44 ± 28.18	<0.001
Uric acid (*μ*mol/L)	389.24 ± 95.69	406.30 ± 103.77	0.05
Cys C (mg/L)	0.88 ± 0.43	0.97 ± 0.39	0.02
Cholesterol (mmol/L)	5.38 ± 1.04	5.38 ± 1.35	0.99
Triglyceride (mmol/L)	1.91 ± 1.16	2.11 ± 1.61	0.09
HDL (mmol/L)	1.17 ± 0.29	1.08 ± 0.26	<0.001
LDL (mmol/L)	3.54 ± 0.78	3.57 ± 1.02	0.65
Fibrosis markers			
FIB-4	1.23 (0.96, 1.63)	1.43 (1.10, 2.09)	<0.001
APRI	0.30 (0.23, 0.40)	0.34 (0.23, 0.53)	0.004

Values are expressed as mean ± standard deviation, no. (%), or median (interquartile range). CAD: coronary artery disease; DM: diabetes mellitus; NLR: neutrophil-to-lymphocyte ratio; ALT: alanine aminotransferase; AST: aspartate aminotransferase; GGT: *γ*-glutamyl transpeptidase; Cys C: Cystatin C; TBIL: total bilirubin; DBIL: direct bilirubin; HDL: high-density lipoprotein; LDL: low-density lipoprotein.

**Table 2:** Association between fibrosis markers, inflammation markers, and CAD in logistic regression models.

Factors	Unadjusted		Adjusted^†^	
	OR (95% CI)	*p* value	OR (95% CI)	*p* value
Fibrosis markers				
FIB‐4 ≤ 2.67	Reference	—	Reference	—
FIB‐4 > 2.67	6.68 (3.19-14.00)	<0.001	5.67 (2.59-12.38)	<0.001
APRI ≤ 1.5	Reference	—	Reference	—
APRI > 1.5	15.30 (3.58-65.42)	<0.001	14.81 (3.24-67.60)	0.001
Inflammation markers				
Neutrophil (10^9^/L)	1.35 (1.23-1.50)	<0.001	1.35 (1.21-1.50)	<0.001
Lymphocyte (10^9^/L)	0.90 (0.71-1.14)	0.369	0.89 (0.69-1.157)	0.389
Monocyte (10^9^/L)	6.32 (2.48-16.12)	<0.001	1.75 (0.62-4.99)	0.293
NLR	1.20 (1.10-1.32)	<0.001	1.18 (1.08-1.30)	<0.001

NLR: neutrophil-to-lymphocyte ratio. ^†^Adjusted: adjusted for gender, age, smoking, hypertension, DM, triglyceride, HDL, and LDL.

**Table 3:** Binary and ordinal regression analysis of the risk for Gensini score in subjects with NAFLD according to fibrosis markers.

Gensini score	Unadjusted		Adjusted†	
	OR (95% CI)	*p* value	OR (95% CI)	*p* value
0 *vs.* >0				
FIB‐4 ≤ 2.67	Reference		Reference	
FIB‐4 > 2.67	6.68 (3.19-14.00)	<0.001	5.67 (2.59-12.38)	<0.001
APRI ≤ 1.5	Reference		Reference	
APRI > 1.5	15.30 (3.58-65.42)	<0.001	14.81 (3.24-67.60)	<0.001
≤9 *vs.* >9				
FIB‐≤2.67	Reference		Reference	
FIB‐4 > 2.67	6.34 (3.34-12.03)	<0.001	5.72 (2.91-11.26)	<0.001
APRI ≤ 1.5	Reference		Reference	
APRI > 1.5	16.23 (4.80-54.85)	<0.001	15.51 (4.38-54.88)	<0.001
<48 *vs*. ≥48				
FIB − 4 ≤ 2.67	Reference		Reference	
FIB − 4 > 2.67	9.06 (4.83-17.02)	<0.001	8.65 (4.37-17.14)	<0.001
APRI ≤ 1.5	Reference		Reference	
APRI > 1.5	12.69 (5.52-29.16)	<0.001	12.00 (4.86-29.61)	<0.001
0 *vs.* 0-9 *vs.* 9-48 *vs.* ≥48				
FIB − 4 ≤ 2.67	Reference		Reference	
FIB − 4 > 2.67	3.33 (2.42-4.60)	<0.001	2.87 (2.05-4.00)	<0.001
APRI ≤ 1.5	Reference		Reference	
APRI > 1.5	4.65 (2.92-7.43)	<0.001	4.08 (2.52-6.62)	<0.001

NLR: neutrophil-to-lymphocyte ratio. Gensini score = 0, non-CAD; 0 < Gensini score ≤ 9, mild coronary artery stenosis; 9 < Gensini score < 48, moderate coronary artery stenosis; Gensini score ≥ 48, severe coronary artery stenosis. ^†^Adjusted: adjusted for gender, age, smoking, hypertension, DM, triglyceride, HDL, and LDL.

**(a):** 

	Association of inflammation markers and HDL with FIB-4				Mediated association with Gensini score				Bootstrapped confidence intervals for the relative indirect effect on Gensini score				
		*β*	SE	*p* value		*β*	SE	*p* value		*β*	SE	Boot LLCI	Boot ULCI
*Model with neutrophil as a mediator*													
Constant	*i1*	4.243	0.120	<0.001	*i2*	6.916	1.541	<0.001					
FIB-4	*a*	0.377	0.045	<0.001	*C*′	4.749	0.581	<0.001	*a*∗*b*	1.286	0.356	0.696	2.080
Mediation of neutrophil					*b*	3.416	0.540	<0.001					
*Model with lymphocyte as a mediator*													
Constant	*i1*	2.235	0.042	<0.001	*i2*	5.293	3.886	0.173					
FIB-4	*a*	-0.072	0.016	<0.001	*C*′	4.749	0.581	<0.001	*a*∗*b*	-0.053	0.143	-0.370	0.205
Mediation of lymphocyte					*b*	0.726	1.596	0.649					
*Model with monocyte as a mediator*													
Constant	*i1*	0.509	0.011	<0.001	*i2*	-5.664	3.308	0.088					
FIB-4	*a*	0.008	0.004	0.056	*C*′	4.749	0.581	<0.001	*a*∗*b*	0.204	0.147	-0.052	0.530
Mediation of monocyte					*b*	24.725	5.779	<0.001					
*Model with NLR as a mediator*													
Constant	*i1*	2.013	0.133	<0.001	*i2*	3.851	1.831	0.036					
FIB-4	*a*	0.442	0.050	<0.001	*C*′	4.749	0.581	<0.001	*a*∗*b*	0.674	0.367	0.112	1.542
Mediation of NLR					*b*	1.523	0.500	0.002					

**(b):** 

	Association of inflammation markers with APRI				Mediated association with Gensini score				Bootstrapped confidence intervals for the relative indirect effect on Gensini score^‡^				
		*β*	*SE*	*p* value		*β*	SE	*p* value		*β*	SE	Boot LLCI	Boot ULCI
*Model with neutrophil as a mediator*													
Constant	*i1*	4.396	0.106	<0.001	*i2*	-5.845	2.746	0.034					
APRI	*a*	1.063	0.122	<0.001	*C*′	12.227	1.584	<0.001	*a*∗*b*	3.692	1.074	1.950	6.147
Mediation of neutrophil					*b*	3.474	0.546	<0.001					
*Model with lymphocyte as a mediator*													
Constant	*i1*	2.182	0.038	<0.001	*i2*	9.237	3.744	0.014					
APRI	*a*	-0.155	0.043	<0.001	*C*′	12.227	1.584	<0.001	*a*∗*b*	-0.013	0.302	-0.683	0.563
Mediation of lymphocyte					*b*	0.085	1.595	0.957					
*Model with monocyte as a mediator*													
Constant	*i1*	0.506	0.010	<0.001	*i2*	-2.079	3.265	0.525					
APRI	*a*	0.037	0.012	0.002	*C*′	12.227	1.584	<0.001	*a*∗*b*	0.834	0.408	0.203	1.804
Mediation of monocyte					*b*	22.754	5.867	<0.001					
*Model with NLR as a mediator*													
Constant	*i1*	2.250	0.120	<0.001	*i2*	5.691	1.769	0.001					
APRI	*a*	1.133	0.137	<0.001	*C*′	12.227	1.584	<0.001	*a*∗*b*	1.880	0.986	0.352	4.144
Mediation of NLR					*b*	1.659	0.498	<0.001					

Boot LLCI/ULCI: bootstrapped B value lower level (LL) or upper level (UL) confidence interval (CI) for the test of mediation, based on 5,000 bootstrap samples. Unstandardized coefficients *β* and standard error (SE) are reported. ^‡^The relative indirect effect is statistically different from zero if the confidence intervals do not include zero.

## Data Availability

The research data used to support the findings of this study are available from the corresponding author upon request.

## Supplementary Materials

Supplementary Materials Table S1: odds ratios (OR) and 95% confidence intervals (CI) for CAD using binary logistic regression. Figure S1: the path diagram of mediation analysis. Figure S2: distribution of inflammation markers according to Gensini score.
