# Supplementary material for: Nonalcoholic Fatty Liver Disease-Associated Liver Fibrosis Is Linked with the Severity of Coronary Artery Disease Mediated by Systemic Inflammation
Source: Dis Markers. 2021 Dec 28;2021:6591784. doi: 10.1155/2021/6591784 (PMC8727161; doi:10.1155/2021/6591784)
Supplement: Supplementary Materials — Table S1: odds ratios (OR) and 95% confidence intervals (CI) for CAD using binary logistic regression. Figure S1: the path diagram of mediation analysis. Figure S2: distribution of inflammation markers according to Gensini score. [file 6591784.f1.docx]

**Table S1 Odds ratios (OR) and 95% confidence intervals (CI) for CAD using binary logistic regression.**

| **Factors** | **Univariate analysis** | | **Multivariable analysis** | |
| --- | --- | --- | --- | --- |
|  | **OR (95%CI)** | ***p* value** | **OR (95%CI)** | ***p* value** |
| Age (year) | 2.64(1.86-3.76) | <0.001 | 1.04(1.02-1.07) | <0.001 |
| Male, *n (*%) | 1.04(1.02-1.05) | <0.001 | 2.36(1.59-3.52) | <0.001 |
| Smoking, *n (*%) | 2.76(1.85-4.117) | <0.001 | - | - |
| Hypertension, *n (*%) | 1.84(1.30-2.62) | 0.001 | 1.59(1.07-2.36) | 0.022 |
| DM, *n (*%) | 1.77(1.22-2.58) | 0.003 | 1.60(1.06-2.41) | 0.027 |
| Platelet (109/L) | 1.00(1.00-1.00) | 0.884 |  |  |
| AST (U/L) | 1.02(1.01-1.03) | <0.001 | 1.01 (1.01-1.03) | <0.001 |
| ALT (U/L) | 1.00(1.00-1.01) | 0.356 |  |  |
| GGT (U/L) | 1.00(1.00-1.00) | 0.873 |  |  |
| Total Protein (g/L) | 0.97(0.94-1.00) | 0.019 | - | - |
| Albumin (g/L) | 0.92(0.88-0.97) | 0.001 | - | - |
| TBIL (μmol/L) | 1.00(0.97-1.03) | 0.98 |  |  |
| DBIL (μmol/L) | 1.02(0.92-1.12) | 0.75 |  |  |
| Creatinine (μmol/L) | 1.02(1.01-1.03) | <0.001 | - | - |
| Uric acid (μmol/L) | 1.00(1.00-1.00) | 0.05 | - | - |
| Cys C (mg/L) | 1.85(1.08-3.17) | 0.024 | - | - |
| Cholesterol (mmol/L) | 1.00(0.87-1.15) | 0.993 |  |  |
| Triglyceride (mmol/L) | 1.11(0.98-1.26) | 0.098 |  |  |
| HDL (mmol/L) | 0.27(0.14-0.53) | <0.001 | 0.36(0.17-0.76) | 0.008 |
| LDL (mmol/L) | 1.05(0.86-1.26) | 0.651 |  |  |

CAD, coronary artery disease; DM, diabetes mellitus; NLR, neutrophil-to-lymphocyte ratio; ALT, alanine aminotransferase; AST, aspartate aminotransferase; GGT, γ-glutamyl transpeptidase; Cys C, Cystatin C; TBIL, total bilirubin; DBIL, direct bilirubin; HDL, high-density lipoprotein; LDL, low-density lipoprotein.

Factors with *p* values <0.05 by univariate analysis were included in the multivariate analysis.


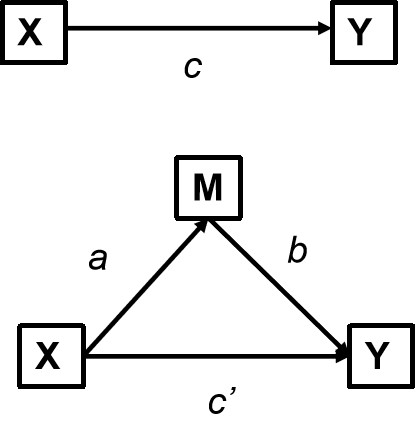


**Figure S1 The path diagram of mediation analysis.**

*c* means the total effect of independent variable *X* on dependent variable *Y*; *a* is the effect of *X* on mediating variable *M*; *b* is the direct effect of mediating variable *M* on dependent variable *Y*; *c’* is the direct effect of *X* on *Y* after adjusting for mediator *M*. The value a*b means the indirect effect.


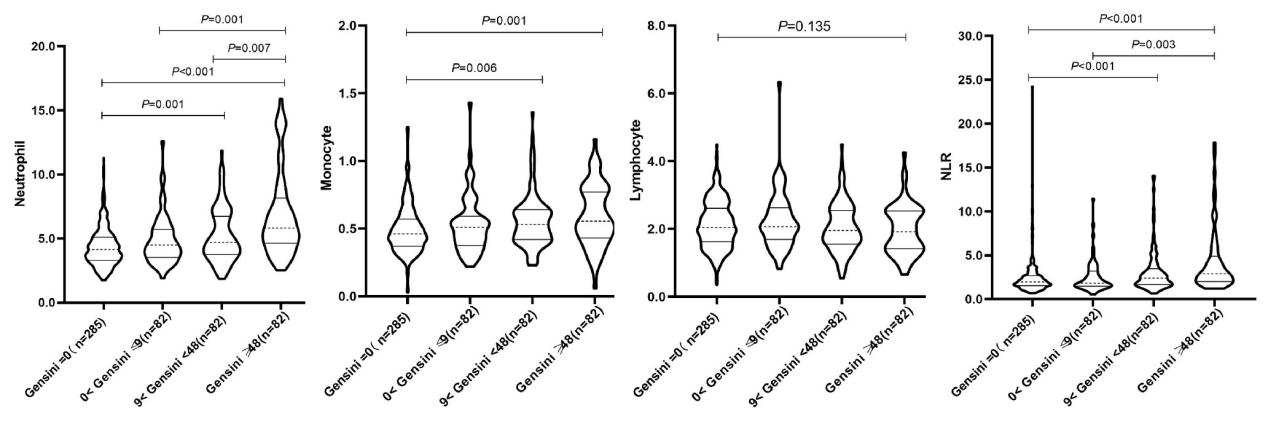


**Figure S2 Distribution of Inflammation Markers According to Gensini Score.**

NLR, neutrophil-to-lymphocyte ratio.

Gensini score=0, non-CAD; 0< Gensini score ≤9, mild coronary artery stenosis; 9< Gensini score <48, moderate coronary artery stenosis; Gensini score ≥48, severe coronary artery stenosis.

The vertical axis represents the distribution of inflammation markers, and the horizontal axis represents different groups according to Gensini score. The data are presented as the median with 25th and 75th percentiles. The dashed lines mean the median. The solid lines below mean 25th percentiles and solid lines above mean 75th percentiles. P values were calculated by Kruskal–Wallis analysis and post hoc analysis.
